# Case Report: Long-term follow-up of multiple giant coronary artery aneurysm associated with multisystem inflammatory syndrome in children

**DOI:** 10.3389/fped.2025.1549321

**Published:** 2025-04-17

**Authors:** Sule Arici, Fatih Alparslan Genc, Gulperi Yagar Keskin, Serafettin Corbacioglu, Ozlem Surekli Karakus, Ayse Inci Yildirim, Metin Sungur, Erkan Tas

**Affiliations:** Department of Pediatric Cardiology, Koşuyolu High Specialization Training and Research Hospital, Istanbul, Türkiye

**Keywords:** case report, multisystem inflammatory syndrome in children (MIS-C), multiple giant coronary artery aneurysms, the long-term results, COVID-19

## Abstract

**Introduction:**

Multisystem inflammatory syndrome in children (MIS-C) is a rare but serious condition that emerged during the COVID-19 pandemic. While most coronary artery abnormalities in MIS-C are transient, the potential for persistent or progressive coronary aneurysms remains unclear. This report presents the long-term follow-up of a pediatric MIS-C case with multiple giant coronary artery aneurysms.

**Case presentation:**

A 4-year-old boy presented with 13 days of persistent fever during the COVID-19 pandemic. MIS-C was diagnosed based on high-grade fever, markedly elevated inflammatory markers, recent SARS-CoV-2 exposure, and coronary artery involvement on echocardiography. The patient showed rapid clinical improvement following treatment with intravenous immunoglobulin, corticosteroids, aspirin, and enoxaparin. Cardiac catheterization at 8 weeks confirmed multiple giant aneurysms in the right and left coronary arteries. He remained asymptomatic and was followed with echocardiography and ECG every 3 months. After 30 months, repeat catheter angiography revealed persistent giant aneurysms, though with slightly reduced dimensions.

**Conclusion:**

This case highlights that multiple giant coronary artery aneurysms associated with MIS-C may persist even after long-term follow-up, despite clinical and laboratory improvement. It underscores the need for extended cardiac monitoring and prolonged antithrombotic therapy in children with severe coronary involvement.

## Introduction

In children, COVID-19 is generally mild; however, in rare cases, it may lead to severe hyperinflammatory conditions. In April 2020, reports from the United Kingdom and Italy described a novel pediatric syndrome resembling incomplete Kawasaki disease (KD) and toxic shock syndrome, later termed multisystem inflammatory syndrome in children (MIS-C) (1).

The diagnostic criteria for MIS-C include persistent fever, elevated inflammatory markers, multisystem involvement, and a history of recent SARS-CoV-2 infection or exposure, with exclusion of other causes (2). Although MIS-C and KD share overlapping features, there are distinct differences, including patient age distribution, inflammatory profile, and cardiac involvement (3). Most MIS-C cases have occurred in older children (≥5 years of age) and adolescents. By contrast, classic KD typically affects infants and young children and has a higher incidence in East Asia and in children of Asian descent (4, 5).

In general, coronary artery involvement in MIS-C is reported to be milder than in KD and tends to resolve within a few weeks (6). However, as in KD, the most troublesome complication in MIS-C is coronary artery dilatation and aneurysm development (5, 6). Since MIS-C is a newly defined disease, long-term outcomes are uncertain.

The aim of this case report is to present the long-term (30-month) follow-up findings of a child diagnosed with MIS-C who developed multiple giant coronary artery aneurysms. We aim to highlight the potential for persistent coronary abnormalities in MIS-C and emphasize the importance of extended cardiac monitoring and antithrombotic therapy in such cases.

## Case presentation

A previously healthy 4-year-old male presented to the emergency department with a 13-day history of persistent fever during the COVID-19 pandemic. He had no prior history of chronic illness, medication use, allergies, or surgical interventions. The patient had not received the COVID-19 vaccine, and there were no missing vaccines in his routine immunization schedule. His family members had experienced symptoms of upper respiratory tract infection due to COVID-19 approximately 1 month prior.

At presentation, he also complained of mild abdominal pain and diarrhea. Vital signs included a temperature of 37.7°C, heart rate of 128 beats/min, blood pressure of 105/65 mmHg, respiratory rate of 24 breaths/min, and oxygen saturation of 99% on room air. Physical examination revealed a weak-appearing child without conjunctival injection, lymphadenopathy, rash, or mucous membrane changes (no injected/fissured lips, injected pharynx, or strawberry tongue). Respiratory and abdominal examinations were unremarkable. Chest radiography showed no infiltrates, and urinalysis was within normal limits.

Laboratory findings revealed marked leukocytosis (21.17 × 10⁹/L) with 24.4% lymphocytes and 62.9% neutrophils, elevated inflammatory markers (CRP 204 mg/dl, IL-6 100 pg/ml), and increased cardiac enzymes (troponin T 0.178 µg/L, NT-proBNP 388 pg/ml). D-dimer (7,340 ng/ml), fibrinogen (462 mg/dl), ferritin (535 ng/ml), and ESR (52 mm/h) were also elevated. The patient was anemic (Hb 9.5 g/dl) and thrombocytosis was present (752 K/µl). A detailed laboratory panel is provided in Table 1.

**Table 1:** Diagnostic and follow-up laboratory test results.

Laboratory parameter	Admission	3 Days from admission	12 Days from admission	8 Weeks follow-up
Leukocytes (WBC) (n.l 4.0–10.0 × 10⁹/L)	21.17 × 10⁹/L	15.11 × 10⁹/L	12.12 × 10⁹/L	9.08 × 10⁹/L
Lymphocytes (n.l. 20%–40%)	24.4%	22.1%	42.8%	35.3%
Neutrophils (n.l. 50%–70%)	62.9%	67.8%	46.6%	55.7%
Platelets (n.l. 150–450 K/µl)	752 K/µl	538 K/µl	335 K/µl	220 K/µl
Hemoglobin (Hgb) (n.l. 13–17 g/dl)	9.5 g/dl	9.8 g/dl	10.7 g/dl	11.9 g/dl
C-reactive protein (n.l. 0–10 mg/dl)	204 mg/dl	155 mg/dl	8	0.6
Interleukin-6 (IL-6) (n.l. <5 pg/ml)	100 pg/ml	1.78 pg/ml	-	-
D-dimer (n.l. <500 ng/ml)	7,340 ng/ml	2,200	440	-
Troponin T (n.l. <0.01 µg/L)	0.178 µg/L	0.162 µg/L	0.076 µg/L	0.006 µg/L
NT-pro BNP (n.l. <125 pg/ml)	388 pg/ml	221 pg/ml	-	80 pg/ml
Ferritin (n.l. 30–400 ng/ml)	535 ng/ml	189 ng/ml	-	-
Fibrinogen (n.l. 200–400 mg/dl)	462 mg/dl	320 mg/dl	272 mg/dl	-
Sedimentation rate (1st hour) (n.l. <20 mm/h)	52 mm/h	33 mm/h	15 mm/hr	-

Due to suspicion of sepsis, empirical intravenous vancomycin and ceftriaxone were started after obtaining blood cultures. The peripheral blood smear was normal, and all infectious serologies and cultures were negative. Antibiotics were discontinued after 72 h upon confirmation of negative cultures.

**Figure 1:**
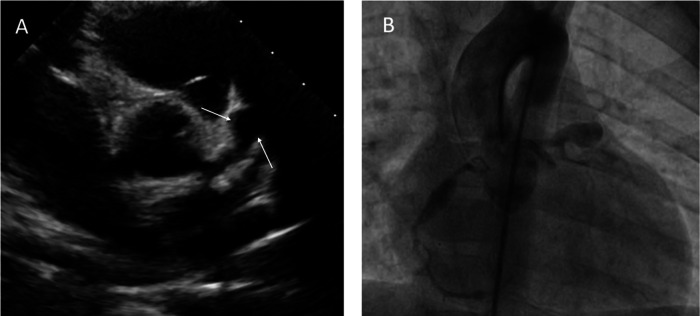
Transthoracic echocardiography showing a giant aneurysm at LAD **(A)**. Coronary angiogram showing multiple giant coronary saccular aneurysm at LAD and RCA **(B)**.

Echocardiographic findings revealed normal left ventricular function (ejection fraction: 65 percent), dilated main left coronary artery (LMCA) 4 mm (*Z* score = 3.03), and left descending coronary artery (LAD) 5.5 mm (*Z* score = 5.69). Additionally, two aneurysms were observed in the LAD, with dimensions of 7 × 11 mm and 5.5 × 7 mm (Figure 1A). No ST-T wave changes were observed in his electrocardiography.

Based on the presence of prolonged fever, markedly elevated inflammatory and cardiac markers, recent exposure to COVID-19, and coronary artery involvement, the diagnosis of MIS-C was made, fulfilling CDC diagnostic criteria.

Treatment with one dose of intravenous immunoglobulin (IVIG) 2 g/kg and one dose of pulse methylprednisolone 30 mg/kg was initiated along with aspirin (30–50 mg/kg/day) and enoxaparin (1 mg/kg/day). His condition dramatically improved with no spike of fever within the next 48 h of illness. Subsequently, inflammatory markers decreased. The daily aspirin dose was reduced (3–5 mg/kg/day). After 1 dose of pulse methylprednisolone was given, methylprednisolone at a dose of 2 mg/kg was continued for up to 7 days. It was then gradually reduced and discontinued within 2 weeks.

The patient underwent cardiac catheterization 8 weeks after diagnosis (Figure 1B). During the coronary angiography, the following measurements and findings were observed. The right coronary artery (RCA) had a diameter of 2.1 mm (*Z* score = 0.16). The LMCA had a diameter of 3.4 mm (*Z* score = 1.64). An aneurysmal dilation with a length of 17 mm and a maximum diameter of 5.5 mm (*Z* score = 8.9) was detected 6 mm distal to the RCA. At a distance of 10 mm from the LMCA, an aneurysm with a length of 22 mm and a diameter of 10.6 mm (*Z* score > 10) was seen in the ostial portion of the LAD. Additionally, a second aneurysmal dilation with a length of 7 mm and a diameter of 7.4 mm (*Z* score > 10) was observed 7 mm distal to the first aneurysm in the continuation of the LAD.

The patient remained asymptomatic and was followed on low-dose aspirin (3–5 mg/kg/day, once daily) and warfarin therapy to prevent thrombotic complications due to multiple giant coronary artery aneurysms. Routine follow-up with echocardiography and ECG was conducted every 3 months.

**Figure 2:**
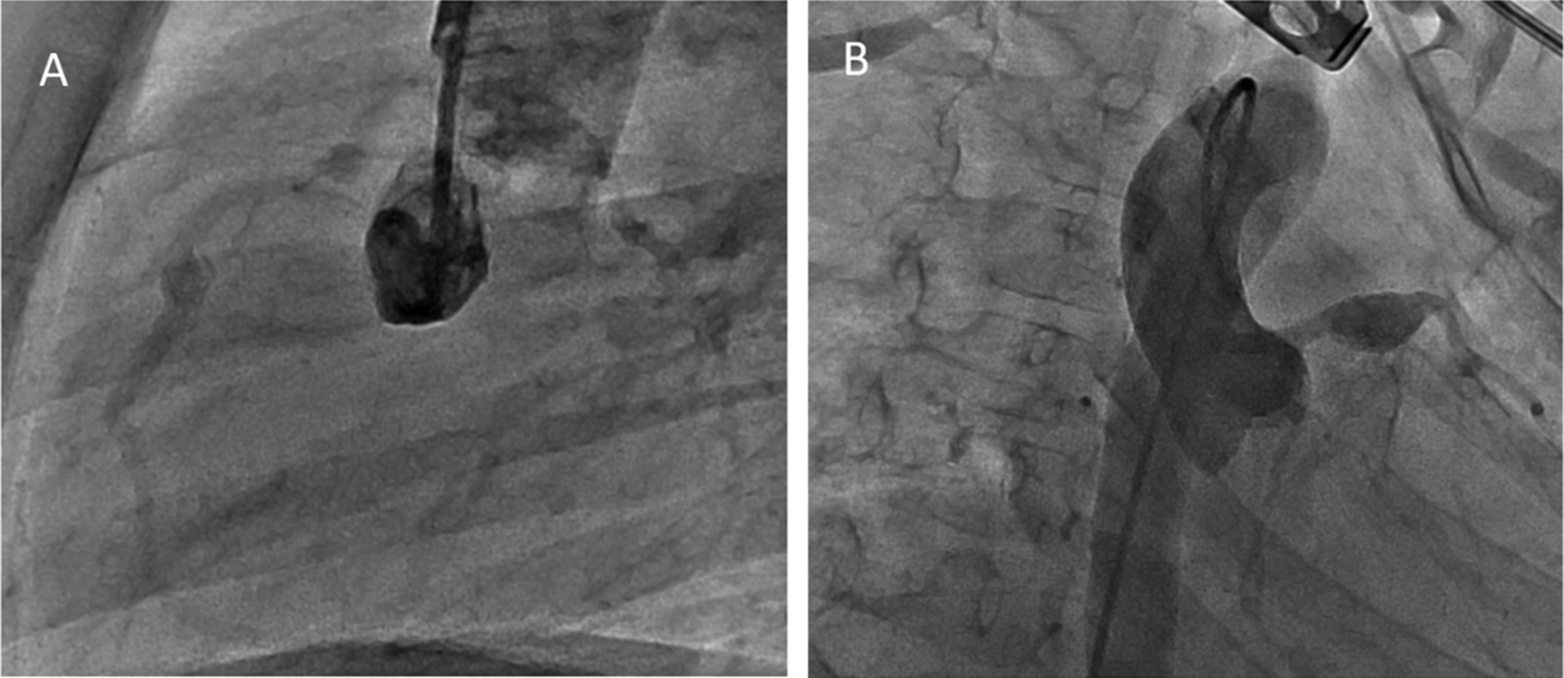
Coronary angiogram showing aright coronary saccular aneurysm involving theproximal and mid-RCA **(A)** and giant aneurysm at LAD **(B)**.

**Figure 3:**
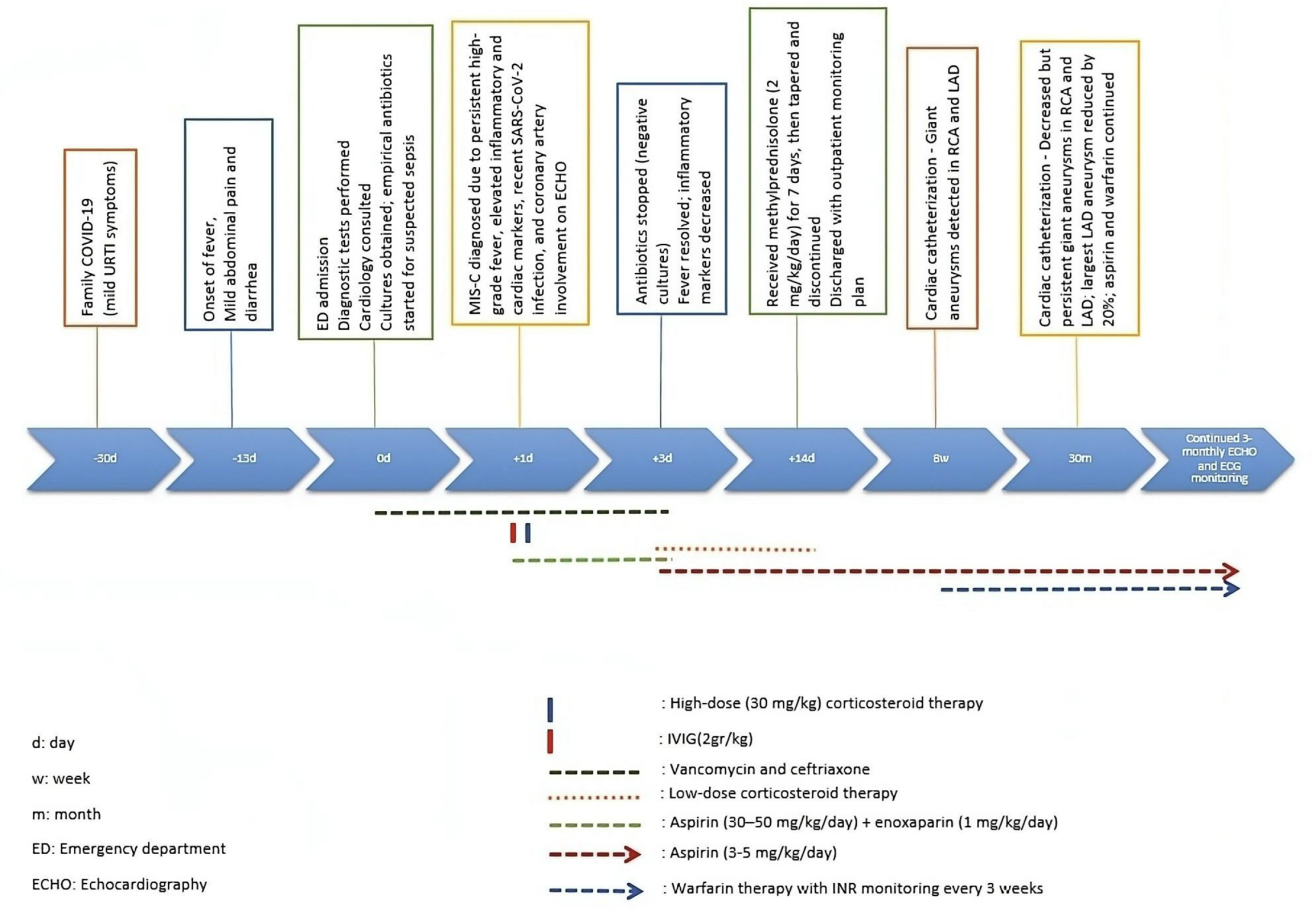
Timeline of symptoms, interventions, and cardiac monitoring.

At 30 months post-diagnosis, catheter coronary angiography was performed (Figure 2). The proximal diameter of the RCA was measured as 1.9 mm (*Z* score = −0.6). An aneurysmal dilation with a length of 5.3 mm and a maximum diameter of 4.17 mm (*Z* score = 5.37) was detected 17 mm distal to the RCA. A small aneurysm with a diameter of 2.6 mm (*Z* score = 2) was observed 11.6 mm distal to the first aneurysm. In the LAD, at a distance of 9.5 mm distal to LMCA, an aneurysm with dimensions of 8.62 mm × 8.56 mm (*Z* score > 10) was observed. The calibrations of the circumflex artery (Cx) and LAD were found to be normal. The LMCA had a diameter of 3.4 mm (*Z* score = 1.88). The largest aneurysm was in the proximal LAD, and its diameter decreased by 20% (Table 2). Although the diameters of coronary aneurysms decreased, aspirin (low dose) and warfarin treatments were continued because of multiple giant aneurysms (Figure 3).

**Table 2:** Coronary artery measurements at various time points.

Time point	Right coronary artery (RCA)	Left main coronary artery (LMCA)	Left anterior descending artery (LAD)
Initial presentation (by echocardiography)	2.1 mm (*Z* score = 0.16)	4 mm (Z score = 3.03)	5.5 mm (*Z* score = 5.69) Aneurysms: 7 × 11 mm, 5.5 × 7 mm
8 Weeks post-diagnosis (by catheter angiography)	2.1 mm (*Z* score = 0.16) Aneurysm: 17 mm (length), 5.5 mm (diameter) (Z score = 8.9) (6 mm distal)	3.4 mm (*Z* score = 1.64)	Aneurysms: 10.6 mm (*Z* score > 10) (22 mm length, ostial portion of LAD), 7.4 mm (*Z* score > 10) (7 mm distal to first aneurysm)
30 Months follow-up (by catheter angiography)	1.9 mm (*Z* score = −0.6) Aneurysm: 5.3 mm (*Z* score = 5.37) (17 mm distal) Aneurysm: 2.6 mm (*Z* score = 2) (11.6 mm distal)	3.4 mm (*Z* score = 1.88)	Aneurysm: 8.62 × 8.56 mm (*Z* score > 10) (9.5 mm distal to LMCA)

A self-assessment checklist has been completed in accordance with the CARE guidelines to ensure accurate and transparent reporting, and is provided as supplementary material (Appendix A).

## Discussion

Multisystem inflammatory syndrome in children (MIS-C) is a novel condition associated with SARS-CoV-2, and its acute manifestations have been well documented. However, long-term outcomes remain uncertain, particularly in cases with severe cardiovascular involvement (5, 7). While coronary artery dilatation in MIS-C is generally considered mild and transient, our case demonstrates that giant coronary artery aneurysms may persist despite early and intensive treatment.

The patient in this report exhibited multiple large aneurysms in both the LAD and RCA, which remained prominent even after 30 months of follow-up. Although echocardiography was performed regularly every 3 months, coronary artery dimensions—especially in distal segments—are more reliably assessed by catheter angiography. Catheter-based imaging provides superior anatomical detail and precise lumen measurements, which are particularly important for evaluating aneurysms in distal coronary segments (8). Therefore, we presented the most accurate follow-up measurements based on the second cardiac catheterization conducted at 30 months after diagnosis. The timeline has been illustrated to provide a clear view of the diagnostic and therapeutic milestones (Figure 3).

Differentiating between MIS-C and Kawasaki disease (KD) remains a diagnostic challenge due to overlapping clinical features. Our patient fulfilled the CDC criteria for MIS-C, including prolonged fever, markedly elevated inflammatory and cardiac markers (CRP, ferritin, D-dimer, troponin, and NT-proBNP), and a recent history of SARS-CoV-2 exposure (2). Although the coronary artery involvement in this case—particularly the presence of multiple giant aneurysms—resembles KD more than the typical MIS-C pattern, the overall clinical and laboratory profile supports a diagnosis of MIS-C.

According to Şener et al., distinguishing KD-like MIS-C from classical KD may be aided by specific laboratory findings: lower lymphocyte and platelet counts, and higher levels of CRP, ferritin, D-dimer, troponin, and BNP are more characteristic of MIS-C (9). Our patient demonstrated most of these features, with the exception of thrombocytosis, which may also occasionally be seen in MIS-C. Additionally, gastrointestinal symptoms such as abdominal pain, vomiting, and diarrhea have been reported more frequently in MIS-C compared to KD (9). The presence of mild abdominal pain and diarrhea at admission in our patient further supports the diagnosis of MIS-C.

These diagnostic uncertainties underline the importance of a comprehensive clinical and laboratory evaluation when managing pediatric patients presenting with systemic inflammation and coronary involvement. Recognizing the subtle differences between MIS-C and KD is essential not only for accurate classification but also for implementing the most appropriate immunomodulatory and antithrombotic management strategies. Furthermore, as emphasized by Haslak et al., the heightened clinical focus on COVID-19 during the pandemic may lead to under-recognition or delayed diagnosis of Kawasaki disease in febrile children (10). This underscores the importance of a balanced and thorough diagnostic approach when encountering overlapping features of MIS-C and KD.

Based on clinical features similar to KD, current treatment for MIS-C includes the addition of glucocorticoids to aspirin and IVIG in moderate-to-severe disease. Infliximab, a tumor necrosis factor antagonist, has been found effective in treating MIS-C patients unresponsive to IVIG (11). Due to widespread coronary artery involvement, we started the patient on pulse steroid, IVIG and aspirin treatment. Although clinical and laboratory improvement was evident, we followed him with aspirin and warfarin treatments in the long term because coronary artery aneurysms persisted.

The mechanism causing coronary aneurysms in MIS-C is unclear. It has been hypothesized that, similar to KD, circulating inflammatory cytokines disrupt the arterial wall (6). Although the coronary abnormalities seen in MIS-C are generally reported to be relatively mild and resolve rapidly, some articles have shown that coronary artery aneurysms can progress even after treatment (12, 13). Maggio et al. reported that coronary artery lesions persisted in 33% of cases during a 10-month follow-up period (12). In our patient, coronary artery involvement was accompanied by giant aneurysms. After 30 months of asymptomatic follow-up, the giant aneurysms continued to have significant dimensions, although their sizes had decreased somewhat.

## Conclusion

This case highlights that multiple giant coronary artery aneurysms associated with MIS-C may persist even after long-term follow-up, despite clinical and laboratory improvement. The findings emphasize the importance of early identification, aggressive immunomodulatory therapy, and close cardiovascular monitoring. Long-term cardiac monitoring is essential to evaluate the course of coronary aneurysms and to guide antithrombotic therapy, especially in patients with persistent or complex lesions.

## Data Availability

The original contributions presented in the study are included in the article/Supplementary Material, further inquiries can be directed to the corresponding author.
